# Measurement of optical reflection and temperature changes after blood occlusion using a wearable device

**DOI:** 10.1038/s41598-020-68152-6

**Published:** 2020-07-13

**Authors:** Jian Gu, Yoko Tomioka, Koichi Kida, Yingyi Xiao, Itsuro Saito, Mutsumi Okazaki, Takao Someya, Masaki Sekino

**Affiliations:** 1 Department of Electrical Engineering and Information Systems, Graduate School of Engineering, The University of Tokyo, Tokyo, Japan; 2 Department of Plastic and Reconstructive Surgery, Graduate School of Medicine, The University of Tokyo, Tokyo, Japan; 3 iMed Japan Inc, Narashino, Japan

**Keywords:** Cardiovascular diseases, Electrical and electronic engineering

## Abstract

Early detection of compromised circulation is essential for postoperative monitoring of free flap. Hourly clinical check-ups such as inspection and palpation still result in a delay in detection. Conversely, optical reflection and temperature measurement are useful alternatives for detecting blood circulation. However, conventional methods that verify ischemia and congestion within a short period have not been reported. In this study, we measured short-term changes in optical reflection and temperature in a rat flap using a wearable flexible sensor probe previously developed in our laboratory. Five ischemia and five congestion groin flap models were measured using a sensor probe and reference devices. Curve fitting was performed on transition signals to evaluate changes in signals and their time constants. The optical reflection signal decreased after venous ligation and increased after arterial ligation. The parameters of the fitted curves indicate a significant difference between congestion and ischemia at *p* < 0.01 (probability value), which was detected within a few minutes after ligation. However, insufficient significance was observed in the temperature signal. Our method gives supporting information to verify ischemia and congestion, and has the potential to rapidly detect compromised circulation.

## Introduction

Tissue transplantation involves surgery to recover tissue defects after cancer excision or injury. Tissue including vasculature is lifted from its original healthy body location and transplanted to the defect location. The tissue, termed a free flap, receives blood supply by anastomosis of arteries and veins at the defect location, restoring circulation to the free flap. As the expected quality of life of patients has been increasing recently, tissue transplantation is more commonly performed in hospitals. However, although the technique has advanced, the risk of flap failure remains 1–9%, depending on the position of the reconstruction^1,2,3,4,5^. The key reason for flap failure is compromised circulation in the blood vessels due to thrombosis^1,6,7^, which occurs in one week after the surgery in most cases. Early salvage surgery soon after the compromised circulation event is essential to increase the rescue rate of tissue^8,9^. Thus, real time detection of tissue circulation is necessary.

Regular postoperative check-ups in hospital involve manual check-ups such as pin prick tests, inspection, and palpation. Pin prick tests monitor blood circulation by examining bleeding from the skin surface. Slow or absent bleeding occurs with arterial occlusion (ischemia), whereas darker bleeding is observed with venous occlusion (congestion). During inspection, a pale flap colour indicates an ischemia event, whereas a cyanotic purple colour represents a congestion event. During palpation, a decrease in skin temperature may suggest ischemia, but temperature change is not significant during congestion^10^. Some groups have reported that hourly check-ups of transplanted tissue are required during the first two postoperative days^7,11,12^, but the time loss between the occurrence of compromised circulation and clinical check-up may reduce the survival rate of the tissue. In addition, conventional manual check-ups consume substantial human resources. Free flap postoperative monitoring devices, such as those used for monitoring the oxygen saturation of arterial blood (SpO_2_)^13^, laser Dopplers^14,15^, near-infrared spectroscopy devices^16^, and pulse wave sensors previously reported by our group^17^, have been developed in recent years to address this issue. These devices have already been used in some hospitals, but their accuracy has not yet been fully demonstrated, and the devices have not replaced medical check-ups by doctors^18^. Therefore, monitoring devices that measure multiple signals could provide a solution to improve the accuracy of this approach.

Measurement of flap colour and temperature conveys similar information as conventional regular medical check-ups. In terms of colour measurement, Prasetyono et al.^19^ photographed and verified the change in rabbit flap colour approximately 30 min after the venous clamp event. However, a more objective and accurate system is required for practical use. Instead, the colour of skin tissue is obtained by measuring optical reflection within the visible light spectrum and evaluated using either the colour spaces recommended by the Commission Internationale de l'Éclairage (CIE) or the colour spectrum. Studies on light absorption by skin tissue showed that haemoglobin and oxyhaemoglobin in blood are the main chromophores under visible light^20,21,22^. Thus, compromised circulation, that is, blockage of the blood vessels, can be detected by measuring optical reflection. Conventional colour measuring devices such as spectrometers, diffuse reflectors, and handheld colorimeters have been used to monitor tissue circulation. Suzuki used a colorimeter to show that the brightness of rat skin decreases 12 h after ischemia and congestion events, but the chromatic quality exhibits minimal changes^23^. Zhu et al. used diffuse reflectance to show reduced optical reflection after both ischemia and congestion occlusion^24^. Their method employs an original score that illustrates significant differences between the control and occlusion groups but less significant differences between the arterial and venous occlusion groups. A subsequent paper also reported less accurate ischemia and congestion classification^25^. Conventional methods measure changes in flap colour with a measurement interval from several minutes to one hour, yet take longer to verify normal, ischemia, and congestion events. Therefore, it is important to measure flap colour transition before and after vascular occlusions for earlier detection.

For temperature measurement, thermography is most commonly used^26,27,28,29,30^. Kraemer et al.^30^ reported the clinical use of thermal cameras after breast reconstruction surgery and found a 2 °C temperature drop in a compromised circulation flap when compared to normal tissue. Chiu et al.^31^ used temperature strips to detect a 2 °C temperature change on an arterial-compromised skin island and a 1 °C temperature change on a venous-compromised skin island. Other clinical studies have confirmed the feasibility of thermography for postoperative flap monitoring, while noting a lack of significant difference between the temperature of the flap and that of the surrounding tissue^10,29,32^. Thus, some researchers only consider this method as supplementary to other measurements because of its unstable flap failure monitoring during the postoperative period^10,33^. Although previous studies have reported a temperature change after flap failure, temperature changes in the flap immediately after compromised circulation have not yet been reported. Thus, early detection of flap failure has not yet been achieved.

In this study, we measured the change in optical reflection and temperature of a rat flap over a short period of time using a wearable flexible sensor probe previously developed in our laboratory^34^. The transition speed as well as the optical reflection and temperature after ischemia and congestion were analysed and compared with those derived from conventional approaches. We then evaluated the feasibility of our proposed short-term transient measurement system.

## Materials and methods

Animal experiments were carried out in accordance with the institutional regulations for animal experiments based on the governmental Guidelines for Proper Conduct of Animal Experiment and Related Activities and were approved by the Institutional Committee of the University of Tokyo (KA14-6).

### Sensing system hardware

The system for measuring the optical reflection and temperature of skin tissue comprised a sensor probe, data transmitter, and tablet monitor, and was previously developed to measure pulse wave signals^34^. The custom-made sensor probe (model number: TTM3-S) and data transmitter (model number: TTM3-T) were designed and fabricated by iMed Japan Inc., and an iPad mini 4 (Apple Inc.) was used as the tablet monitor.

#### Sensor probe

**Figure 1:**
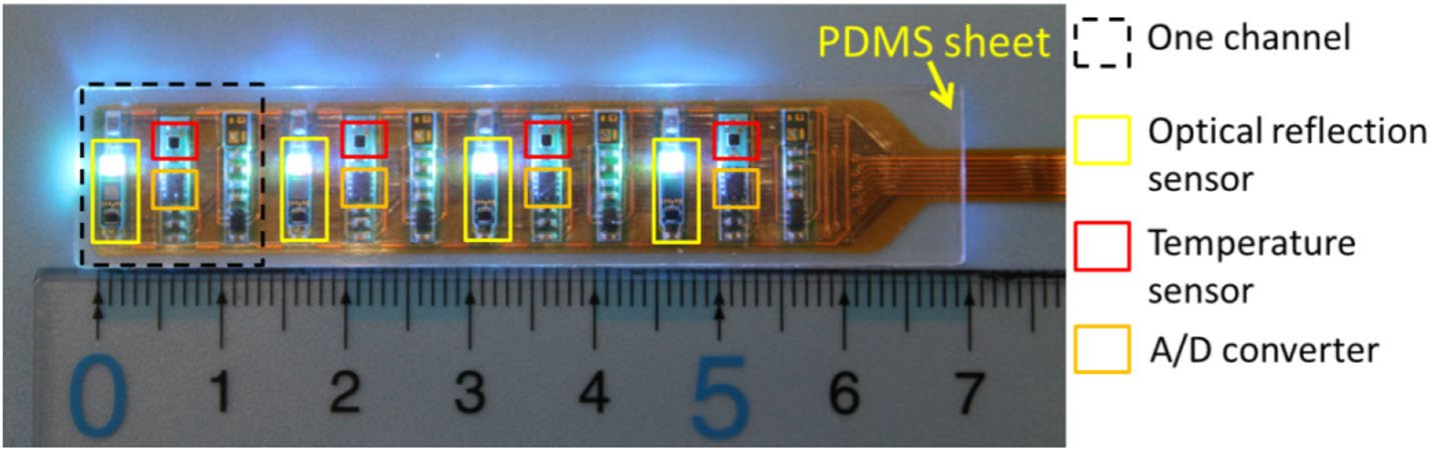
Structure of the wearable sensor probe. Locations of the colour sensors, temperature sensors, and A/D converters are marked by yellow, red, and orange boxes, respectively. All sensors are mounted on a polyimide substrate. The PDMS sheet was placed on the sensor probe to create a flat surface (yellow arrow).

Optical reflection sensors, temperature sensors, and A/D converters were implemented on a 60-μm-thick flexible substrate, as shown in Fig. 1. In the figure, four optical reflection sensors are marked by yellow boxes, temperature sensors are marked by red boxes, and A/D converters are marked by orange boxes. The dashed black box defines the area of one channel on the probe. The four channels in Fig. 1 are termed channel 1, 2, 3, and 4, from left to right. The optical reflection sensors on the probe were compact colour sensors (P12347, Hamamatsu Photonics, Japan), which were composed of RGB LED light sources, an Inter-Integrated Circuit (I^2^C) chip, and a colour sensing area. The colour sensing area represents the optical reflection component covering red (590–660 nm), green (490–600 nm), blue (395–530 nm), and infrared (630–1,000 nm) light. The signal output of the colour sensor was digital data that could be read using an I^2^C bus. The temperature sensors (LMT70YFQ, Texas Instruments) yielded analogue voltage data and transmitted the data to A/D converters ((ADS1115IRUG, Texas Instruments). The converted digital data was also transmitted through the I^2^C bus. Sensors and electrical devices were soldered onto a polyimide substrate using surface mount techniques developed by Taiyo-Kogyo Corporation (Japan). The resolution of the sensor was 15 mm, which was determined by the interval between sensor channels. The distance between optical reflection and temperature sensors in channels was 14.5 mm. The sensors were 1 mm thick after implementation, but other implanted chips were thinner (0.2–0.7 mm), causing an uneven surface. To produce a flat sensing surface, we designed a flexible transparent sheet to fill the space between the sensor probe and skin surface, as shown by the yellow arrow in Fig. 1. The sheet was made from polydimethylsiloxane (PDMS) with a mixture of Silpot 184 and Silpot CAT (Toray, Japan) at a ratio of 20:1. The materials were mixed in a mould and heated at 100 °C for 1 h. After peeling off the mixture from the mould, the sheet was completed. A sensor probe and a transparent sheet were sandwiched between two medical transparent films (Tegaderm 3 M, USA), and then the sensor could be attached to the target position together with the film. Tegaderm 3 M film is used practically in clinical trials. Details of the sensor attachment can be found in our previous report^34^.

#### Data transmitter

An 8-bit microcontroller (C8051F410, Silicon Labs) was used to receive the optical reflection and temperature signal on the I^2^C bus and control the operation of the LED and measurement timing. The system stored red, green, blue, and infrared reflectance and temperature data every 5 min. Temperature signals of each channel were measured for 0.1 s, whereas optical reflection signals of each channel were measured for 0.4 s.

### Rat model of compromised circulation

To evaluate the effectiveness of our system on two types of compromised circulation, we conducted ischemia and congestion rat model experiments. Five male Wistar rats (mean ± standard deviation of body weight: 397 $\pm$ 17 g, Nos. 1–5) were used in the ischemia model experiment and five male Wistar rats (mean ± standard deviation of body weight: 448 $\pm$ 36 g, Nos. 6–10) were used in the congestion model experiment. The left groin flaps of the rats were lifted up and only connected to the body using the inferior epigastric arteries and veins. Arterial occlusion events were provoked on the groin flap by ligation of the connecting artery using a nylon suture (10–0 U.S.P, Keisei Medical Industrial, Japan). Venous occlusion events were provoked on the groin flap by ligation of the connecting vein. Reperfusion was provoked in both models by releasing the ligature. The size of the left groin flap was 3.5 cm $\times$ 7 cm. Normal tissue to the right of the flap was employed as control tissue. A thermal insulation sheet (DS-2L, FUSO-Kasei, Japan) with a waterproof layer was sutured between the flap and the abdomen to suppress heat transfer from the body of the rat. The sensor probe was attached perpendicularly to the body of the rat across the groin flap and control tissue using a transparent medical film (Tegaderm 3M, USA). Channels 1 and 2 of the sensor measured signals from the groin flap, whereas channels 3 and 4 measured signals from the control tissue.

### Experimental measurements

The measurements of both compromised circulation models started approximately 30 min after preparation of the rat models. The experiments followed three steps: before ligation, after ligation, and the reperfusion period. In the first step, normal rat circulation was measured for 5 min, then vascular occlusion was provoked by ligation. The ligation of vessels took 10–40 s to complete. In the second step, compromised circulation of groin flap was measured for 10 min after the starting time of ligation. Then, the ligature was released. In the last step, reperfusion of the flap was measured for 5 min after releasing the ligature. At the beginning of the first step, the end of the last step, and 5 min after ligation, reference photographs, optical reflection measurements, and temperature measurements were obtained. The reference optical reflection was recorded using a colour meter (TES125, Satotech, Japan) and the reference temperature was recorded by thermography (InfReC Thermo GEAR G120EX, Avio, Japan).

### Analysis of reference data

The colour meter was aimed at evaluating the groin flap and control tissue and measurements were taken five times on both sides. The sensor probe was attached to the bottom half of the flap area and the colour meter was located in the upper half to avoid the influence of light source from the probe. The outputs of the colour meter were expressed by the XYZ colorimetric system, where the Y value expresses the brightness of the colour. The average values of X, Y, and Z from five locations were calculated as the reference values.

The thermograph was placed above the abdomen of the rat for each experiment and the resulting thermogram was analysed using accessary software (InfReC Analyzer NS9500 Standard). Two rectangular areas measuring 40 a.u $\times$ 50 a.u were selected: one on the groin flap (area A) and one on the control tissue (area B). The total size of the thermogram was 320 a.u $\times$ 240 a.u. The average values of each box were calculated as the reference values.

### Analysis of sensor probe data

The optical reflection components on the sensor probe measuring the reflected light volume and light volume outputs were transformed to XYZ values by applying the equation proposed by Hamamatsu Photonics^35^:

1 $$\left[\begin{array}{c}X\\ Y\\ Z\end{array}\right]=\left[\begin{array}{cc}\begin{array}{cc}\begin{array}{c}0.35\\ 0.12\\ -0.02\end{array}& \begin{array}{c}0.53\\ 1.16\\ -0.12\end{array}\end{array}& \begin{array}{cc}\begin{array}{c}0.22\\ 0.05\\ 1.67\end{array}& \begin{array}{c}-1.32\\ -1.61\\ -1.83\end{array}\end{array}\end{array}\right]\left[\begin{array}{c}\begin{array}{c}R\\ G\end{array}\\ \begin{array}{c}B\\ IR\end{array}\end{array}\right]$$

The average XYZ values from the three steps were calculated for each rat. For the brightness signal (Y), the transition from the starting time of ligation to 5 min after ligation was fitted using an exponential curve ($y={A}_{0}+A*{e}^{-\frac{t-{t}_{0}}{\tau }}$) to evaluate the change soon after compromised circulation, where $A$ represents the amplitude of the brightness change and $\tau$ represents the time constant of the transition. Similarly, for temperature data, the average values from the three steps were calculated. The transition of temperature was fitted using the same exponential curve, and parameters $A$ and $\tau$ were calculated to assess the amplitude and time constant of the temperature change, respectively.

### Statistical analysis

The average value and standard deviation of brightness, temperature, $A,$ and $\tau$ were calculated for the five rats in each rat model. Signals from channel 1 and channel 2 were used for the groin flap data set, and signals from channel 3 and channel 4 were used as the control tissue data set. These data sets were classified into the “before ligation” group, “after ligation” group, and “reperfusion” group, accordingly. The groups were analysed for statistical significance using the paired two-sided t-test. The differences were assessed with an α level of 0.05.

## Results

### Visual flap changes in the congestion and ischemia models

**Figure 2:**
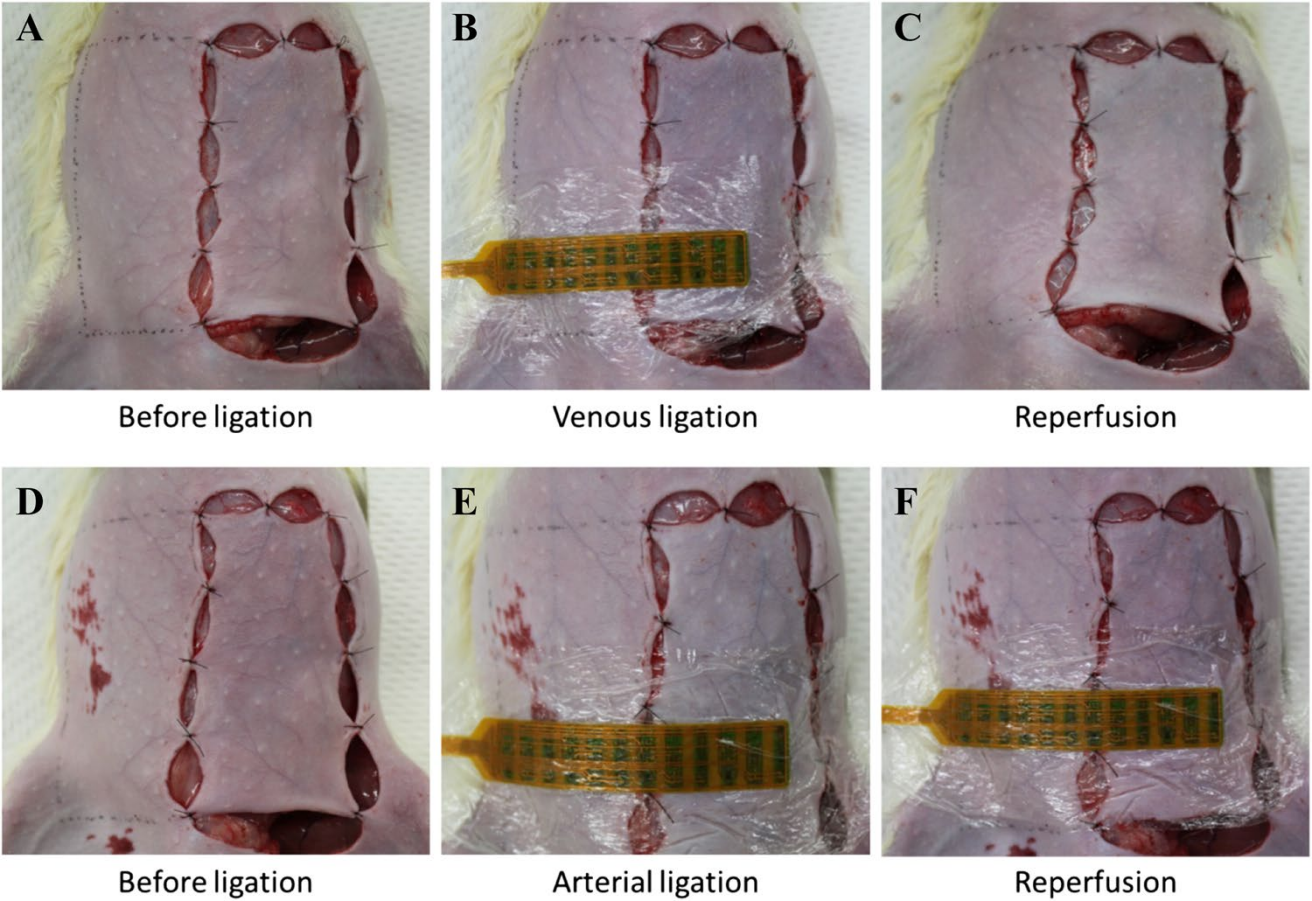
Provocation of compromised circulation on the rat’s groin flap model. The photographs show the colour of the flap before venous ligation (**A**); 5 min after venous ligation (**B**); during reperfusion (**C**); before arterial ligation (**D**); 5 min after arterial ligation (**E**); and during reperfusion (**F**). The flap colour in (**B**) is dark purple, whereas that in (**E**) is pale. The flap colour returned to normal for both models in (**C**) and (**F**).

In our experiments, the flaps with venous occlusion and arterial occlusion did not become necrotic because of the short-term nature of the ligation. The photographs in Fig. 2A–C show the three typical steps in a congestion experiment. The groin flap and control tissue during the first step had a similar colour, indicating normal blood circulation 30 min after lifting the flap (Fig. 2A). The groin flap then became dark purple 5 min after venous ligation (Fig. 2B), exhibiting a clear colour difference from the control tissue. The groin flap returned to a similar colour as that of the control tissue during reperfusion (Fig. 2C). Figure 2D–F show the same three steps for an ischemia experiment. The colour of the groin flap became pale 5 min after arterial ligation, in contrast to that of the control tissue (Fig. 2E). A similar colour was observed for the groin flap and control tissue in Fig. 2D and F, indicating normal blood circulation and reperfusion. These results demonstrate the conventional qualitative clinical check-ups, and the observed changes in colour are consistent with empirical findings in clinical cases of impaired circulation.

### Optical reflection in the congestion and ischemia models

**Figure 3:**
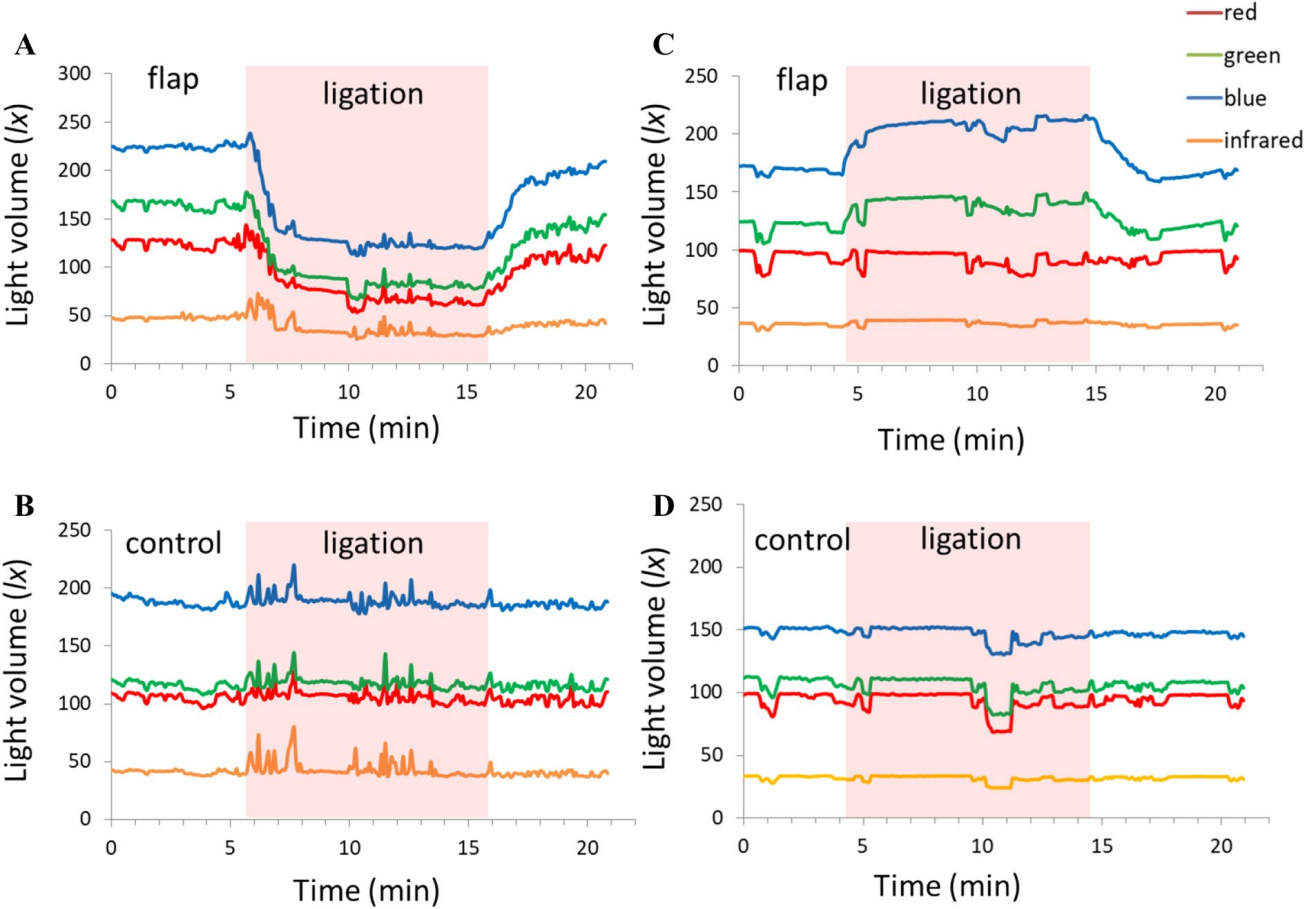
Raw optical reflection data of the compromised circulation model. Red, green, blue, and infrared light volumes measured on (**A**) groin flaps in the congestion model; (**B**) control tissue in the congestion model; (**C**) groin flaps in the ischemia model; and (**D**) control tissue in the ischemia model. The recorded ligation time is marked by the red shaded zone from approximately 5–15 min.

The raw optical reflection signal of rat Nos.1–5 was measured in congestion experiment, and a sample signal transition of rat No.1 is shown in Fig. 3A,B. Changes in the red, green, blue, and infrared components on the groin flap are shown in Fig. 3A. The light volume of red, green, and blue components decreased sharply 1 min after venous ligation, then gradually decreased with continued ligation. The light volume was then recovered to approximately the original levels after releasing the ligature. The infrared component did not change during the congestion experiment. On the other hand, the light volumes of all components in the control tissue showed almost no change during the congestion experiment (Fig. 3B). Fluctuations in the raw data were due to noise from the operation or reference measurements.

The raw optical reflection signal of rat Nos. 6–10 was measured in ischemia experiment, and a sample signal transition of rat No.6 is shown in Fig. 3C,D. Figure 3C plots the change in all optical reflection components on the groin flap. The light volume of green and blue components increased sharply in the first minute after arterial ligation, then gradually increased with continued ligation. The light volumes of both components recovered to approximately the original levels after releasing the ligature. The red and infrared components did not change during the ischemia experiment. The light volumes of all components in the control tissue showed almost no change during the ischemia experiment (Fig. 3D).

**Figure 4:**
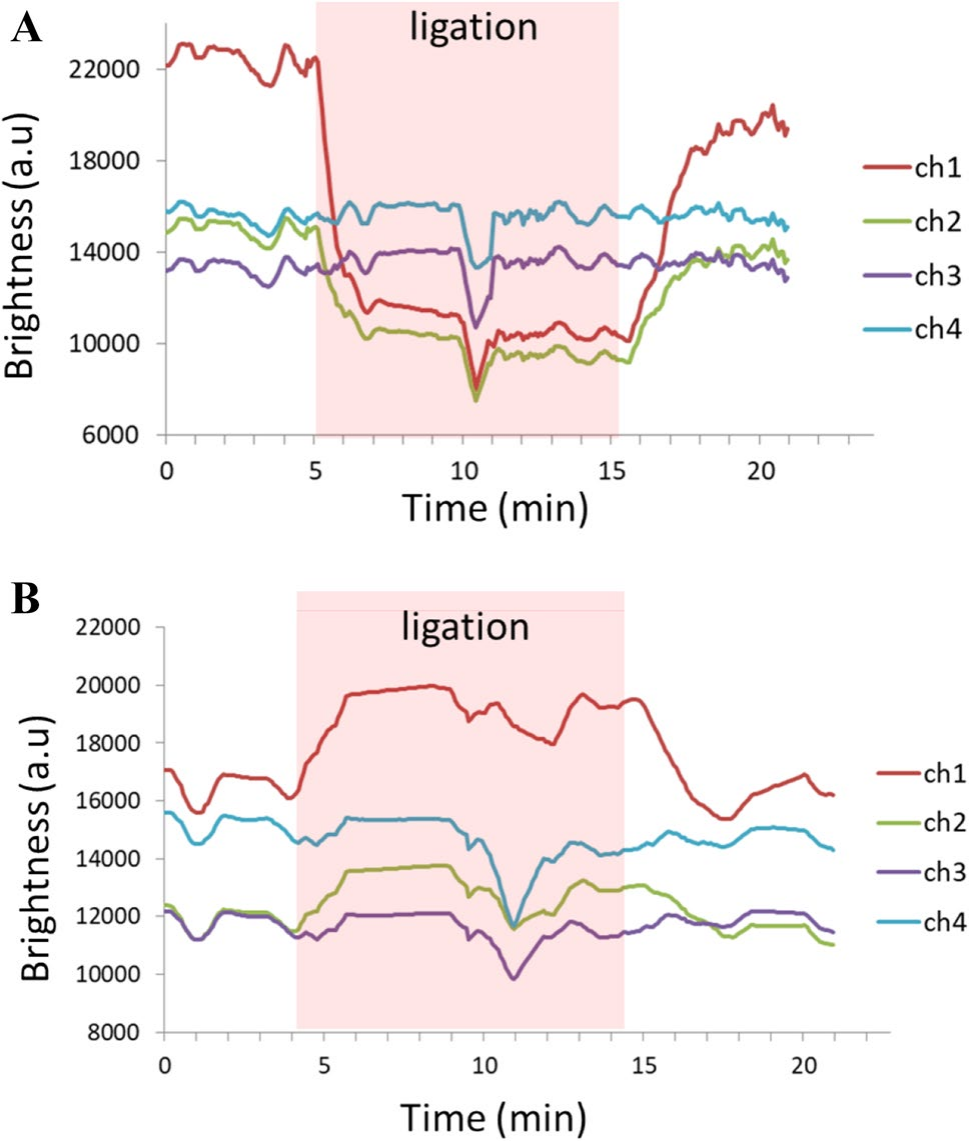
Calculated brightness of the compromised circulation model. Data from the four sensor channels for (**A**) the congestion model and (**B**) the ischemia model corresponded to the raw data in Fig. 3. Channels 1 and 2 measured the groin flap and channels 3 and 4 measured the control tissue. The recorded ligation time is marked by the red shaded zone.

The colour components were transformed into the XYZ system as the output. The brightness (Y) obtained from the groin flap (channel 1 and channel 2) and control tissue (channel 3 and channel 4) in the congestion experiment is plotted in Fig. 4A. Groin flap Y decreased during ligation, whereas control tissue Y remained stable. The patterns of brightness change between sensing channels were similar, but the amplitudes were different (channel 1: approximately 10,000 a.u, channel 2: approximately 5,000 a.u) owing to the measurement location. A sudden brightness change at 10 min was caused by the reference measurement. The brightness (Y) obtained during the ischemia experiment is plotted in Fig. 4B. Again, groin flap Y increased during ligation, whereas control tissue Y remained stable. The amplitudes of brightness change during arterial ligation were less than those during venous ligation (channel 1: approximately 4,000 a.u, channel 2: approximately 2000 a.u).

### Statistical results

**Figure 5:**
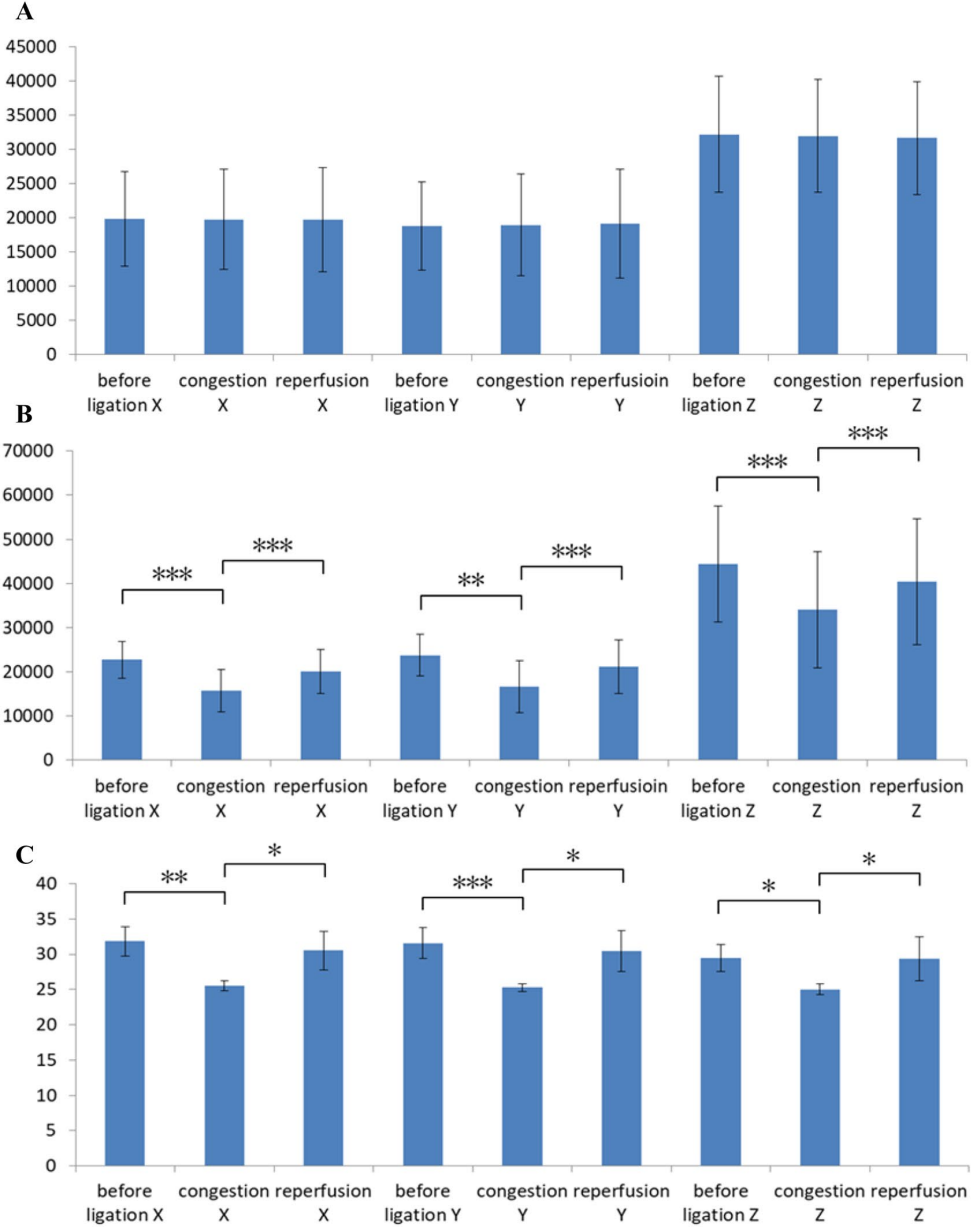
Statistical analysis of XYZ values for the congestion model, showing the average and standard deviations. (**A**) Control tissue measured by the sensor probe; (**B**) groin flap measured by the sensor probe; and (**C**) groin flap measured by the colour meter (*: *p* < 0.05, **: *p* < 0.01, ***: *p* < 0.005).

**Figure 6:**
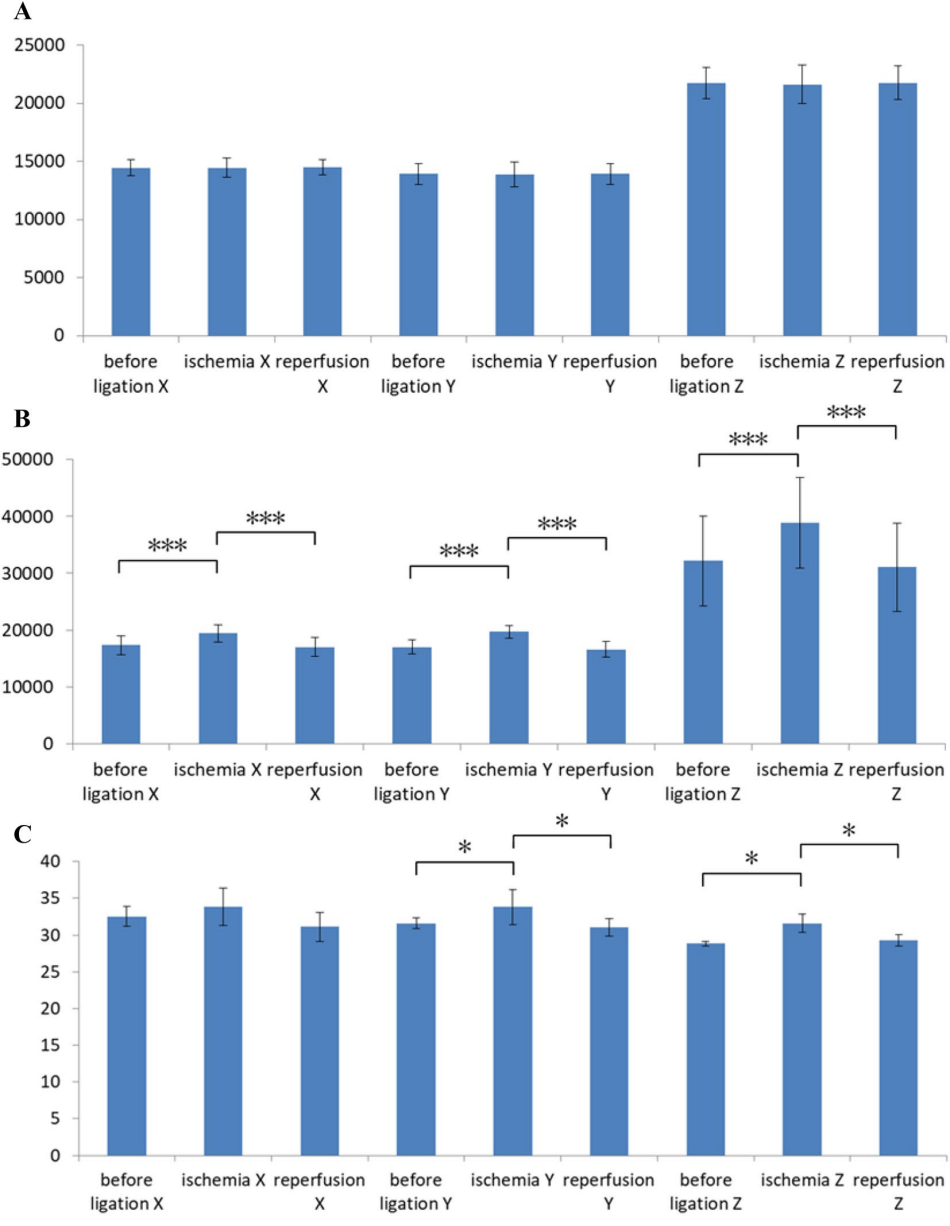
Statistical analysis of XYZ values for the ischemia model, showing the average and standard deviations. (**A**) Control tissue measured by the sensor probe; (B) groin flap measured by the sensor probe; and (**C**) groin flap measured by the colour meter (*: *p* < 0.05, **: *p* < 0.01, ***: *p* < 0.005).

**Figure 7:**
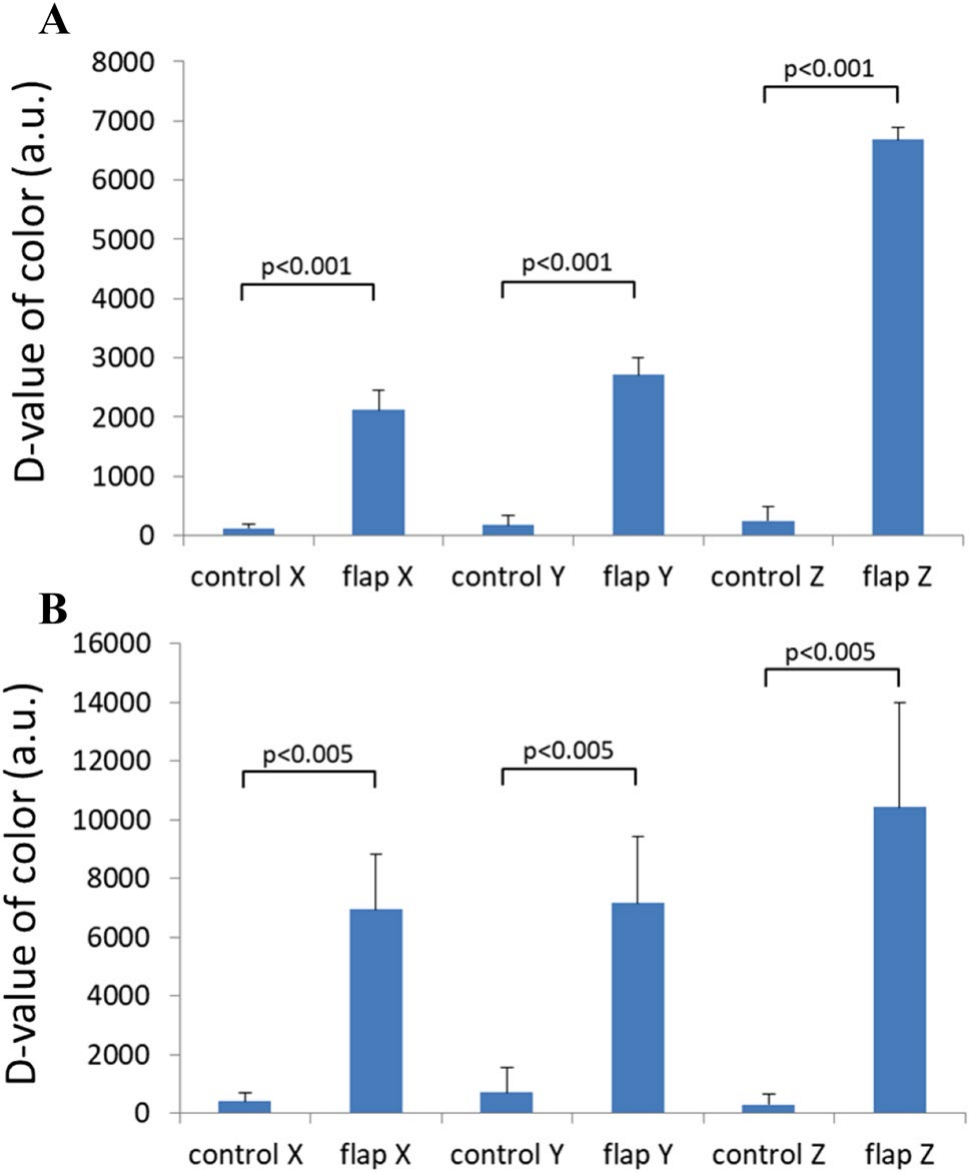
Statistical analysis of XYZ values between the control side and the flap side during ligations, showing the average and standard deviations. (**A**) Congestion model; (**B**) ischemia model.

The bar charts in Fig. 5 show the average values and standard deviations of rats in the congestion experiment for the control flap data set, groin flap data set, and reference groin flap data set. The output signals X, Y, and Z are depicted in the three columns on the left, the middle three columns, and the three columns on the right of each bar chart, respectively. For each signal (X, Y, or Z), the before ligation group, congestion group, and reperfusion group are shown from left to right. The output in Fig. 5A indicates no significant difference among the control tissue data set. Conversely, significant differences were found between groups for all XYZ signals of the flap (Fig. 5B). The average value and standard deviation of X and Y were approximately the same. The p values of before ligation and reperfusion groups were less than 0.01 and 0.005, respectively. The Z signal was larger than the other two and showed comparable p values to X and Y. The reference data set in Fig. 5C provides the corresponding results obtained by the colour meter. The scatter of each bar, which indicates the variance of colour of rat skin, is very different between individuals. However, significant difference is observed in Fig. 5B because a paired sample t-test was used to determine whether the mean difference between two sets of observations is zero. This statistical test enabled us to detect the difference between signals obtained before ligation, after venous ligation, and during reperfusion, even if there were large individual variabilities in baseline signals. The bar charts in Fig. 6 show the average values and standard deviations of rats in the ischemia experiment. Similarly, significant differences were confirmed between groups for all XYZ signals (Fig. 6B). The p values of before ligation and reperfusion groups were all smaller than 0.005. Figure 7A shows the difference in colour between the control and flap side after venous ligation, whereas Fig. 7B shows the difference after arterial ligation. The p values of control and flap groups were all smaller than 0.005, and the average values are larger than the variance values. Thus, the threshold of flow change can be predicted at approximately 1,500 a.u. XYZ measurements.

### Signal transition during compromised circulation events

**Figure 8:**
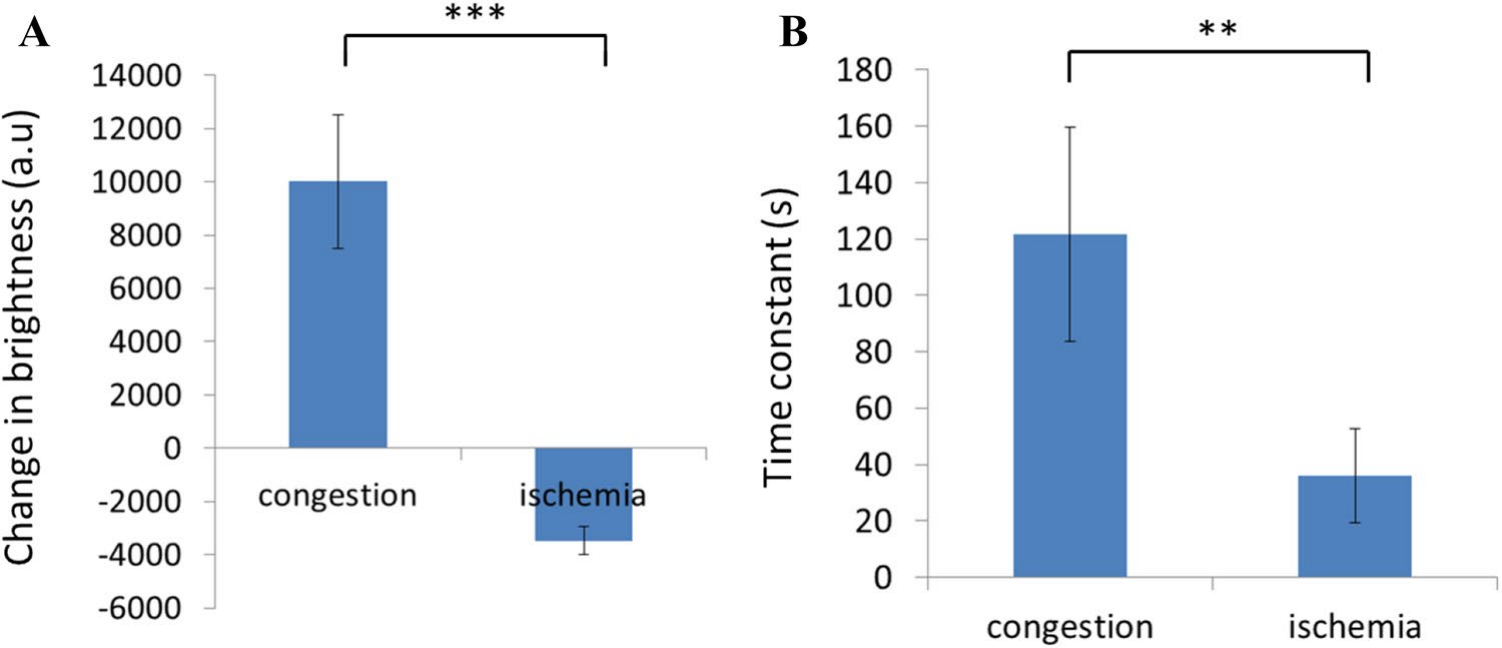
Statistical analysis of changes in brightness and their time constants for the ischemia and congestion groups. The average values and standard deviations of curve fitting parameters representing the change in brightness ($A$) and time constant ($\tau$) are plotted in bar charts (**A**) and (**B**), respectively (**: *p* < 0.01, ***: *p* < 0.005).

The curve fitting results of optical reflection for all rats in congestion and ischemia experiments are shown in Fig. 8. The average values and standard deviations of parameter $A$ in Fig. 8A suggest a significant difference between congestion and ischemia with a p value smaller than 0.005. The positive value of $A$ implies a decreasing trend during congestion, whereas the negative value implies an increasing trend during ischemia. The average values and standard deviations of parameter $\tau$ in Fig. 8B suggest a significant difference between congestion and ischemia with a p value smaller than 0.01. The time constant of congestion transition was approximately 120 s, which was longer than that of ischemia transition (approximately 30 s).

### Temperature measurement for the compromised circulation model

**Figure 9:**
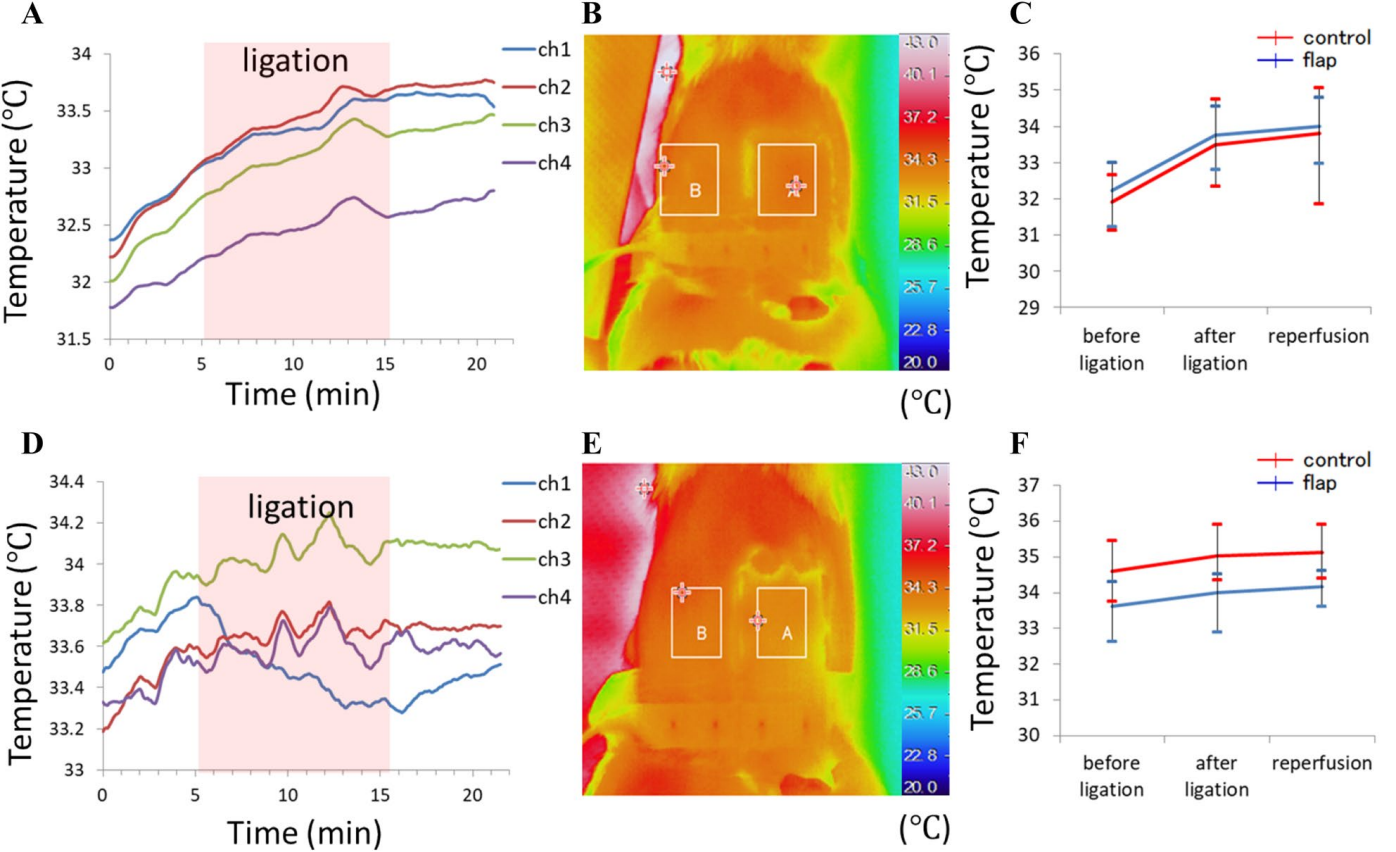
Temperature measurement for the ischemia model. (**A**) Raw data of rat No. 6 measured by the probe; (**B**) reference thermal graph for rat No. 6 after ligation; (**C**) average and standard deviation of temperature for the boxes in (**B**) (box A: groin flap, box B: control tissue). (**D**) Raw data of rat No. 9 measured by the probe; (**E**) reference thermal graph for rat No. 9 after ligation; (**F**) average and standard deviation of temperature for the boxes in (**E**). The recorded ligation time is marked by the red shaded zone.

**Figure 10:**
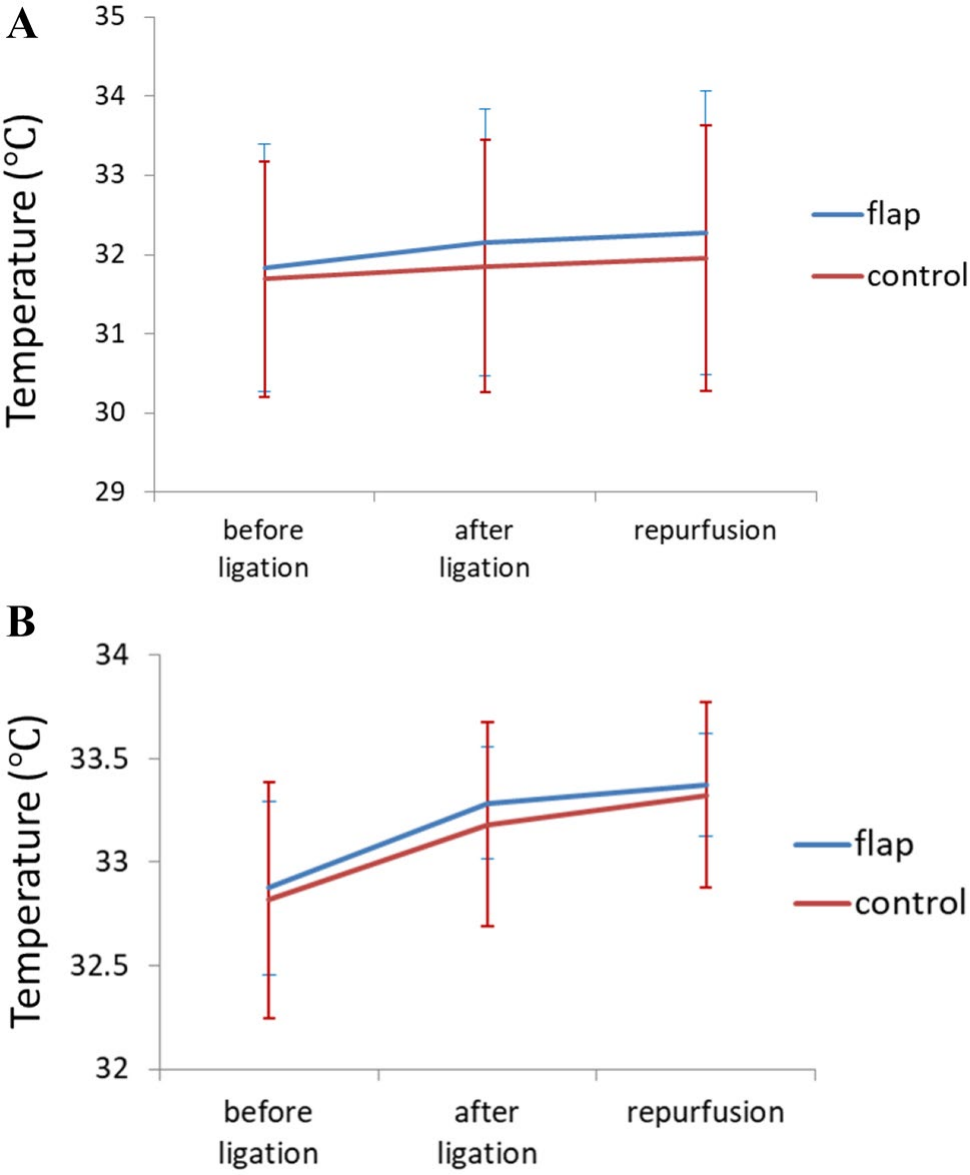
Statistical analysis of temperature for the (**A**) congestion and (**B**) ischemia model showing the average and standard deviation for the five rats. No significant difference was found between groups.

The temperature signals measured by the sensor probe and reference thermography are illustrated in Fig. 9 for rat No. 6 (Fig. 9A–C) and rat No. 9 (Fig. 9D–F). All other rats showed similar results as rat No. 6. The temperature curves from four channels in Fig. 9A indicate the corresponding change during ischemia experiment. The thermal graph obtained 5 min after arterial ligation indicates no obvious temperature difference between the groin flap and control tissue (Fig. 9B). The average value and standard deviation of temperature in box A and box B of Fig. 9B are displayed in Fig. 9C. The reference temperature of the groin flap showed similar changes as the control tissue, with a distribution of $\pm 1 ^\circ \mathrm{C}$. The unique case of rat No. 9 plotted in Fig. 9D shows that the temperature measured by channel 1 decreased from 33.8$^\circ \mathrm{C}$ to 33.3$^\circ \mathrm{C}$ within 10 min after ligation, then recovered to 33.5$^\circ \mathrm{C}$ 5 min after reperfusion. The temperature measured by all other channels remained stable. However, the thermal graph and calculated reference temperature in Fig. 9E,F did not detect this transition after ligation. The high temperature zone to the left of the rat corresponds to the heat pad used to maintain the body temperature of the rat. Figure 10 shows the average values and standard deviations for the five rats measured by sensor probe during each experimental step of congestion and ischemia model. No significant difference was found between the before ligation and after ligation groups of the after ligation and reperfusion groups.

## Discussion

### Mechanism of compromised circulation measurement by sensor probe

The optical reflection signal transition after arterial or venous occlusion is attributable to the change in arterial or venous blood volume, respectively. The structure of the sensor consists of a pair of LEDs and photo transistor at a 4-mm interval, which formed an arch-shaped detection area inside the target tissue. The incident visible light passing through the arterial blood, venous blood and melanin decayed according to the Beer–Lambert Law (${I}_{x}={I}_{0}{e}^{-{\mu }_{a}L}$), where ${I}_{x}$ and ${I}_{0}$ are the received and incident light intensity, respectively, ${\mu }_{a}$ is the absorption coefficient of tissue components, and $L$ is the mean path length of light passing through the corresponding components. The change in blood volume is the major influence on the transition signal, and parameters ${\mu }_{a}$ and $L$ depend on the wavelength of the incident light according to reports from multiple authors^22,36^. Absorption by melanin and other static tissue components had minor effects because neither parameter changes rapidly after vascular occlusion. Thus, the time related term during compromised circulation was the mean path length of arterial and venous blood, which is written as follows:

2 $${I}_{x}\left(\lambda ,t\right)={I}_{0}*{\varepsilon }_{static}*\mathrm{exp}\left\{-\left[{{\mu }_{artery}\left(\lambda \right){L}_{artery}\left(\lambda ,t\right)+\mu }_{vein}\left(\lambda \right){L}_{vein}\left(\lambda ,t\right)\right]\right\},$$

where ${\varepsilon }_{static}$ is the light reduction that would not be affected by blood circulation, ${\mu }_{artery}$ and ${\mu }_{vein}$ are the absorption coefficients of arterial and venous blood, respectively, and ${L}_{artery}$ and ${L}_{vein}$ are the mean path lengths of light in each vasculature, respectively. For blue and green wavelengths, the absorption coefficients of haemoglobin and oxyhaemoglobin were approximately the same and more sensitive than those of red light. For the red wavelength, the volume of haemoglobin was higher than that of oxyhaemoglobin. Thus, the absorption coefficient of arterial blood was lower than that of venous blood because the former contains more oxyhaemoglobin. On the other hand, the penetration depth of green and blue light could only cover the capillary bed at the epidermis and dermis, whereas that of red light could reach parts of the veins, arteries, and venous plexus. When venous occlusion occurred, venous blood filled up most of the capillary bed and vein area near the flap. The venous blood volume in the detection area increased; therefore, the mean path length of venous blood increased. According to formula 2, optical reflection of red, green, and blue components decreased after venous occlusion. When arterial occlusion occurred, blood supply in the capillary bed and arteries was blocked. The arterial blood volume in the detection area decreased; therefore, the mean path length of arterial blood decreased. Optical reflection of green and blue components increased after arterial occlusion. However, reduced blood absorption extended the penetration depth of red light to deeper venous plexus, which reduced optical reflection. Consequently, optical reflection of red light did not change substantially compared to other wavelengths.

The fitting results of brightness value indicated that the amplitude of the transition after venous ligation was greater than that of arterial ligation. The former manipulation allowed the supplied blood to be retained in the expanded vein and capillary area, such that the blood volume at the local tissue area would be several times more than the normal capacity of capillaries. The latter manipulation stopped blood supply, such that the decrease in blood volume in the local tissue was approximately equal to the normal capacity. Moreover, the time constant of the congestion transition was longer than that of ischemia transition. This is possibly because filling of capillary blood lasted for a considerable amount of time owing to tension from the capillary wall, but capillary blood was lost immediately when arterial blood supply was stopped.

### Effect on coloured skin

The optical signals, including visible light and infrared, used in our system would be influenced by the coloured skin because of the absorption by melanin. This is one of the reasons for using a fitting result to detect impaired circulation rather than using a threshold to distinguish raw data. Although we did not discuss the relationship between the penetration depth of light and coloured skin in this paper, detecting the transition of brightness would reduce the effect of baseline skin colour.

### Short-term temperature change after arterial occlusion

Short-term temperature measurement failed to show significant differences after ligation. Although there was no reproducibility in the experiment, rat No. 9 indicated a possible temperature transition after ligation, which was not detected by intermittent approaches. A reduction of 0.5 °C within 10 min after arterial occlusion suggested that the typical temperature change of 2 °C reported by other authors^10^ could be more rapid in some cases. Thus, detection of temperature changes can provide earlier evaluation of skin circulation. Channel 1 and channel 2 on the probe both measured the groin flap of rat No. 9, but channel 2 showed no temperature change after ligation. This indicates that a temperature distribution exists on the flap. For instance, the thermal graph in Fig. 8E shows that the peripheral flap area is cooler than the central flap area. Therefore, calculating the average temperature in a certain area could blur transition during compromised circulation. Thus, multipoint sensing of flap temperature could be more meaningful.

Both thermography and our sensor probe affect rat tissue circulation detection due to environmental artefacts. The aim of applying thermography as a reference is to verify whether 1 °C reduction after compromised circulation reported in other clinical studies is also valid in rat experiment^31^. However, the clinical reports showed that temperature change occurs in some cases after compromised circulation but not in all cases, suggesting that the results of our animal model are consistent with the findings of previous clinical studies. Thus, by combining colour and temperature measurement with other existing wearable methods, such as photoplethysmography sensors^17^, compromised circulation cases can be picked up with higher sensitivity, thereby improving the reliability of the detecting system. Furthermore, temperature is expected to provide supporting information to evaluate the progression of compromised circulation.

### Difference between the proposed measurement system and conventional methods

In this study, we measured acute changes instead of the conventional chronic changes in compromised circulation using wearable sensors. Our wearable monitoring system has potential to improve the user experience for both patient and medical workers, but requires compact sensors^17,34^. Conventional methods using spectrometry measure optical reflection more precisely by scanning multiple wavelengths. Our results show that using red, green, and blue wavelengths is meaningful to evaluate ischemia and congestion. In addition, conventional measurements have occasionally reported a decrease in optical reflection after ischemia and congestion^23,24,25^, which depends on the duration after the onset of impaired blood flow and on the animal model. Conventional inspection based on manual check-up provides qualitative evaluations; for instance, flap colours inspected by doctors are “pale” and “dark purple” after ischemia and congestion, respectively. On the other hand, our device provides a quantitative measurement of this evaluation. The increase and decrease in brightness after ischemia and congestion, respectively, are consistent with empirical findings shared by medical staff.

Many contactless monitoring or imaging devices have been reported. Kamshilin et al.^37^ employed blood-flow monitoring systems in upper limbs. Yu et al.^38,39^ developed noncontact speckle contrast diffuse correlation tomography. Contactless optical solutions have many advantages such as device safety, imaging function, and high accuracy. On the other hand, camera-based optical blood-flow methods are not suitable for long-term monitoring because the action of patients is limited as long as monitoring continues. Contact methods such as our wearable device can monitor patients without limiting their actions. In addition, flexible sensor probes have another advantage as it can measure circulation at any position apart from the limb or forehead. Thus, our device is very important in continuous measurement particularly for flap observation during the postoperative period, which usually lasts for one week. We have attempted alternative ways to detect flap circulation for validation such as ICG and laser Doppler. However, only inferior epigastric artery and vein are connected to the groin flap in our rat model, such that local blood-flow is obviously zero after ligation. It is relatively hard to detect the signal using these imaging methods.

### Placement of the sensor probe in clinical practice

In clinical application, colour and temperature change is easier to measure if the sensor is attached along the supply artery and vein or nearby branches. In addition, it is possible to use several probes simultaneously to cover the entire flap area for colour and temperature mapping. According to the results, optical reflection signal could detect the blood volume change in the capillary layer. In our rat model, the blood volume in supply vessels changed the most after ligation. Therefore, the signal of the channel near supply vessels shows a steeper change than those of other channels. On the other hand, the average temperature of the skin tissue did not change significantly soon after compromised circulation because of thermal equilibration between small vessels (with diameters below 0.2 mm) and surrounding tissues. For rat circulation in our model, only inferior epigastric arteries and veins (with diameters of 0.15–0.25 mm) can be thermally significant according to the local structures. We attempted to attach at least one channel on the tissue where inferior epigastric vessels were the main culprit vessel. Temperature change due to circulation manipulation was captured; however, there was no significant result in the paired t-test. In clinical applications, the average diameters of human vessels are larger (diameters of artery and vein are 4–5 mm), such that it is possible to measure temperature change.

### Future work

The observed transition in optical reflection values in our rat flap experiments suggest that compromised circulation can be evaluated more rapidly using curve fitting. In the future, we plan to establish an algorithm to verify normal circulation, ischemia, and congestion. The feasibility of the measurement system and algorithm will be assessed by a clinical study. In terms of temperature measurement, we intend to continue the development of the sensor probe and determine a suitable layout and number of sensors required to trace temperature transition after vascular occlusion.

The correlation between colour change and temperature change obtained using this wearable probe is another interesting approach to improve the accuracy of detecting compromised circulation, and determining a threshold is also important for making judgement in clinical use. Since measurement in animals and human varies, and a 10-min vessel ligation of a healthy volunteer is not safe, a clinical study is necessary to find the threshold for detecting compromised circulation as well as investigate the correlation between colour and temperature in detail. Processing of background noise is essential in the construction of a judging algorithm. The above research questions are currently being investigated, and will be published in another paper.

## Summary

Acute changes of rat flap optical reflection and temperature were measured over a short period using a wearable flexible sensor probe previously developed in our laboratory. The optical reflection signal and calculated brightness decreased after venous ligation and increased after arterial ligation. A significant difference between normal circulation and compromised circulation was found in the optical reflection signal, but not in the temperature signal. The optical reflection of congestion exhibited a larger change than that of ischemia, whereas the ischemia event exhibited more rapid changes. Our measurement provided objective data during tissue circulation monitoring, which could assist medical staff to verify ischemia and congestion soon after blood occlusion. This measurement system can be applied to postoperative monitoring as a supplementary device.

## Supplementary information

Supplementary information
