# Supplementary material for: Measurement of optical reflection and temperature changes after blood occlusion using a wearable device
Source: Sci Rep. 2020 Jul 13;10:11491. doi: 10.1038/s41598-020-68152-6 (PMC7359365; doi:10.1038/s41598-020-68152-6)
Supplement: Supplementary file 1 — Supplementary information [file 41598_2020_68152_MOESM1_ESM.docx]

Measurement of optical reflection and temperature changes after blood occlusion using a wearable device

Jian Gu^a^, Yoko Tomioka^b^, Koichi Kida^a^, Yingyi Xiao^a^, Itsuro Saito^c^, Mutsumi Okazaki^b^, Takao Someya^a^, Masaki Sekino^a^

a *Department of Electrical Engineering and Information Systems, Graduate School of Engineering, The University of Tokyo, Japan*

b *Department of Plastic and Reconstructive Surgery, Graduate School of Medicine, The University of Tokyo, Japan*

c *iMed Japan Inc, Japan*

Validation of skin flap viability was performed to demonstrate the condition of rat flap after manipulations in our experiments. Four types of manipulations were observed: 1) To ligate artery continuously; 2) To ligate artery for 10 mins and then release the ligature; 3) To ligate vein continuously; 4) To ligate vein for 10 mins and then release the ligature. The observation lasted for 1 week after each manipulation, and the rats were reared for 1 week. Photographs of the flap condition are shown in Fig. S1. For manipulation 1, the colour of the flap became pale after arterial ligation. Necrosis of flap was in progress from day 2 to the last day. For manipulation 2, obvious necrosis could not be observed in the following week. However, compared with the control side, furs did not grow on the flap side, which indicates that acute ischemia manipulation may injure the tissue to some extent. For manipulation 3, the duration of observation was not long because the nylon thread we used to ligate the vein loosened from the vessel after the rat was allowed to move freely in the cage. For manipulation 4, the colour of the flap became dark purple after arterial ligation. Furs grew on both the control side and flap side from day 2 to the last day, which indicates that acute congestion manipulation did not damage the tissue.

| 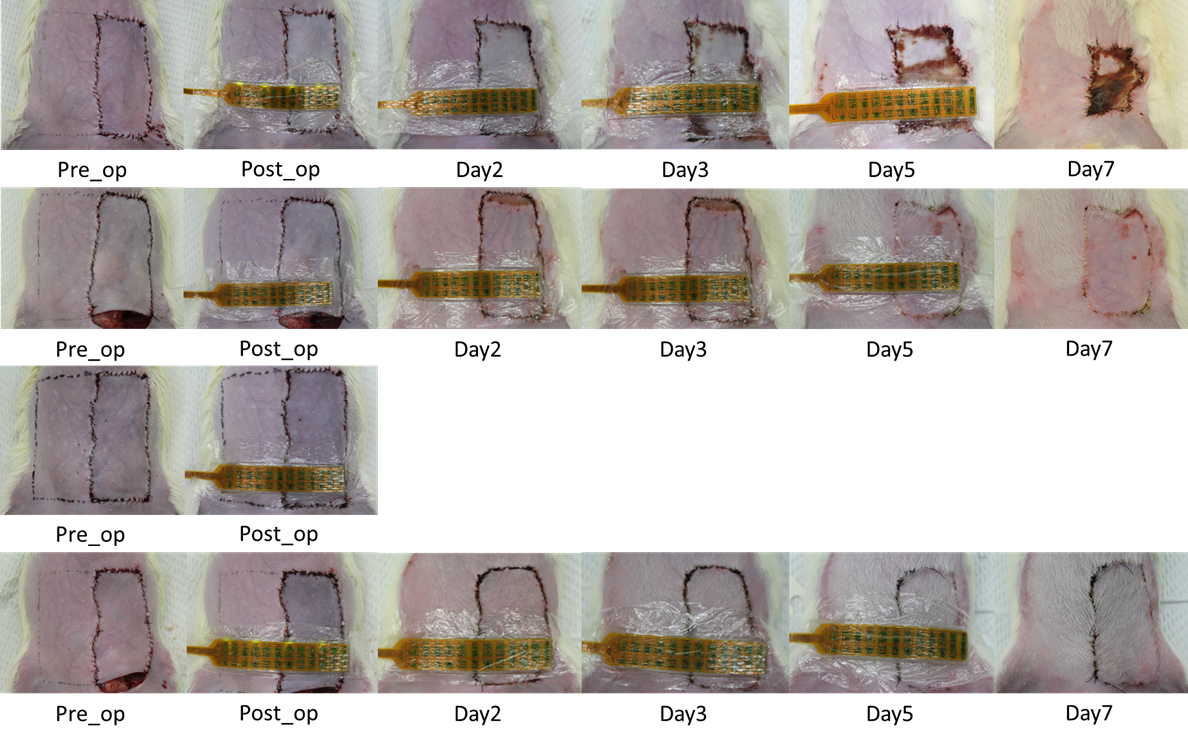 |
| --- |
| Fig.S1 Flap condition after manipulations. Results of manipulations 1 to 4 are shown from the top row to the bottom row. Pre_op and Post_op represent the pre-operation and post-operation period on day 1, respectively. Venous ligation failed for more than one day, only two photographs are shown. |
